# Biocompatibility of Titania Nanotube Coatings Enriched with Silver Nanograins by Chemical Vapor Deposition

**DOI:** 10.3390/nano7090274

**Published:** 2017-09-15

**Authors:** Piotr Piszczek, Żaneta Lewandowska, Aleksandra Radtke, Tomasz Jędrzejewski, Wiesław Kozak, Beata Sadowska, Magdalena Szubka, Ewa Talik, Fabrizio Fiori

**Affiliations:** 1 Faculty of Chemistry, Nicolaus Copernicus University in Toruń, ul. Gagarina 7, 87-100 Toruń, Poland; zaneta.muchewicz@gmail.com (Ż.L.); aradtke@umk.pl (A.R.); 2 Nano-implant Ltd., NIP 9562314777, Gagarina 5, 87-100 Toruń, Poland; 3 Faculty of Biology and Environment Protection, Nicolaus Copernicus University in Toruń, ul. Lwowska 1, 87-100 Toruń, Poland; tomaszj@umk.pl (T.J.); wkozak@umk.pl (W.K.); 4 Faculty of Biology and Environmental Protection, University of Lódź, ul. S. Banacha 12/16, 90-237 Łódź, Poland; beata.sadowska@biol.uni.lodz.pl (B.S.); Ewa.Talik@us.edu.pl (E.T.); 5 August Chełkowski Institute of Physics, University of Silesia, ul. Uniwersytecka 4, 40-007 Katowice, Poland; magdalena.szubka@us.edu.pl; 6 Di.S.C.O.—Sezione di Biochimica, Biologia e Fisica, Università Politecnica delle Marche, 60131 Ancona, Italy; f.fiori@univpm.it

**Keywords:** titania nanotube coatings, silver nanograins, surface morphology, biointegration properties, immunological activity

## Abstract

Bioactivity investigations of titania nanotube (TNT) coatings enriched with silver nanograins (TNT/Ag) have been carried out. TNT/Ag nanocomposite materials were produced by combining the electrochemical anodization and chemical vapor deposition methods. Fabricated coatings were characterized by scanning electron microscopy (SEM), X-ray photoelectron spectroscopy (XPS), and Raman spectroscopy. The release effect of silver ions from TNT/Ag composites immersed in bodily fluids, has been studied using inductively coupled plasma mass spectrometry (ICP-MS). The metabolic activity assay (MTT) was applied to determine the L929 murine fibroblasts adhesion and proliferation on the surface of TNT/Ag coatings. Moreover, the results of immunoassays (using peripheral blood mononuclear cells—PBMCs isolated from rats) allowed the estimation of the immunological activity of TNT/Ag surface materials. Antibacterial activity of TNT/Ag coatings with different morphological and structural features was estimated against two *Staphylococcus aureus* strains (ATCC 29213 and H9). The TNT/Ag nanocomposite layers produced revealed a good biocompatibility promoting the fibroblast adhesion and proliferation. A desirable anti-biofilm activity against the *S. aureus* reference strain was mainly noticed for these TiO_2_ nanotube coatings, which contain dispersed Ag nanograins deposited on their surface.

## 1. Introduction

The low toxicity of titanium and its alloys, as well as their mechanical properties and corrosion resistance, has meant that these materials are widely used in the production of implants for dentistry, maxillofacial surgery, orthopedics, and so on [1,2]. The quality of implants significantly depends on their surface properties and influences their tissue integration properties and osseointegration [3]. An analysis of previous reports revealed that the increased surface roughness of implants, and thereby an increased area, led to improved cell migration and attachment to the implant, as well as to an enhanced osseointegration process [4,5]. Therefore, the surface treatment of implants (i.e., mechanical, physical, and chemical) is used to increase cell adhesion and proliferation, to accelerate the biointegration processes, and to improve surface wettability [6,7,8,9]. Earlier works revealed that the fabrication of the titania nanotubes coating (TNT) on the surface of the titanium substrate significantly increased implant biointegration [1,10,11]. Moreover, a noticeable influence of nanotube diameter and its crystallinity on the adhesion and proliferation of fibroblasts on the surface of TNT layers was observed [10]. A significant issue, which should be solved during the construction of modern implants, is the necessity to provide anti-infection activity. This is especially important in the use of Ti/Ti alloy implants in the oral and craniofacial environment. These properties of implants can be achieved by the addition of biostatic/biocidal components. Antibiotics may be used for this purpose. However, problems associated with the antibiotic resistance of bacteria and their short-term effect mean that their effectiveness may be weak [12]. Another way is to enrich TiO_2_-based layers with silver nanoparticles [13,14,15]. Silver reveals strong antibacterial and antifungal properties through the interaction with bacterial and fungal proteins and enzymes, as well as causing the structural damage of the bacterial and fungal cell wall and membrane [13,16,17,18]. Therefore, silver exhibits great potential for being applied in the fabrication of metal implant coatings in order to prevent biomaterial-related infections. Earlier reports revealed that magnetron sputtering, physical vapor deposition (PVD), sol-gel, and chemical reduction were usually applied to the production of silver-incorporated TNT (TNT/Ag) nanocomposites [16,17,18,19,20]. The results of these investigations proved that suitable antibacterial activity was observed for the dispersed Ag grains of a diameter below 40 nm [14,21,22,23,24]. The application of chemical vapor deposition (CVD) to enrich TNT coatings with dispersed silver nanograins is rather rare, despite the fact that this method provides a controlled growth of silver grains and adequate dispersion on the substrate surface (both flat as well as 3D substrates) [14,25]. It may be necessary to solve the problems associated with the precursor choice and the optimization of CVD conditions, which will allow the deposition of Ag grains on the surface of TNT coatings, without changes to their structure and morphology. Moreover, the fabricated TNT/Ag composite coatings should possess optimal biointegration properties and should simultaneously exhibit suitable antibacterial activity.

The results of our works on the use of the CVD method to incorporate silver grains into titania nanotube coatings (TNT) are discussed in the presented paper. We have focused on the optimization of the CVD process in order to deposit dispersed Ag grains on the surface of TNT substrates, and on the estimation of antibacterial properties and biointegration activity of the produced coatings. The obtained results should be helpful in the design and in the construction of implants, which will be characterized both by suitable biointegration properties and also by anti-inflammation activity.

## 2. Results

### 2.1. Fabrication and Characterization of TNT/Ag Nanocomposite Coatings

The studied titania/silver nanocomposite coatings were obtained using a two-stage procedure. The first step was connected with titania nanotube production and the second one with the enrichment of nanotubes with silver nanograins. The titania nanotube (TNT) layers have been produced by the electrochemical anodization of the titanium foil at selected potentials, i.e., 4 V (TNT4), 6 V (TNT6), and 18 V (TNT18). X-ray diffraction (XRD) and Raman spectroscopy studies confirmed that the TNT4, TNT6, and TNT18 samples were amorphous. The thermal stability of the produced coatings has been determined by an analysis of Raman spectra and SEM images of samples heated between 303 and 573 K. The obtained results revealed that the structure of TNT4 and TNT6 did not change, whereas the TNT layers produced at 18 V (TNT18) exhibited an anatase structure (Figure S1). Simultaneously, the tubular architecture of heated samples was unchanged at this temperature range. In the next stage, the TNT4, TNT6, and TNT18 coatings were enriched with the Ag nanograins using the CVD method. In all deposition experiments, Ag(OOCC_2_F_5_) has been applied as a metallic silver precursor. The deposition conditions are presented in detail in the Materials and Methods section of this article. An analysis of XRD patterns confirmed the presence of Ag grains on the surface of Ti (reference sample) and TNT substrates (Figure S1). The results of CVD experiments revealed that the formation of uniform films, composed of dispersed silver grains, only occurred for the use of the precursor weight *m* = 5 and 10 mg. Mass differences before and after the CVD process of TNT/Ag samples suggest the formation of coatings containing ca. 1 wt% and 1.5 wt% of silver grains. The analysis of XPS data (Table 1, Figure S2) shows peaks at binding energy regions 458.9–459.5 eV and 464.7–465.3 eV, which can be attributed to Ti(2p_3/2_) and Ti(2p_1/2_) core levels of Ti^4+^ [26]. The composite O(1s) peaks are distributed into three peaks at 530.3–530.5, 531.7–532.0, and 532.7–533.0 eV, assigned to O^2−^ in the Ti–O bond, surface –OH groups (these groups change into ·OH free radicals in the photogeneration of electron-hole pairs), and surface H_2_O molecules, respectively (Table 1 and Figure S2) [27]. The H_2_O/TiO_2_ molar ratio (calculated as the area ratio of peaks assigned to H_2_O and O^2−^) is 0.2, 0.1, and 0.6 for TNT4/Ag, TNT6/Ag, and TNT18/Ag, respectively. Peaks at 368.0–368.8 eV (Ag 3d_5/2_) and 373.9–374.9 eV (Ag 3d_3/2_), with the splitting of the 3d doublet of 5.9–6.1 eV, indicate the formation of silver clusters [28,29].

**Figure 1:**
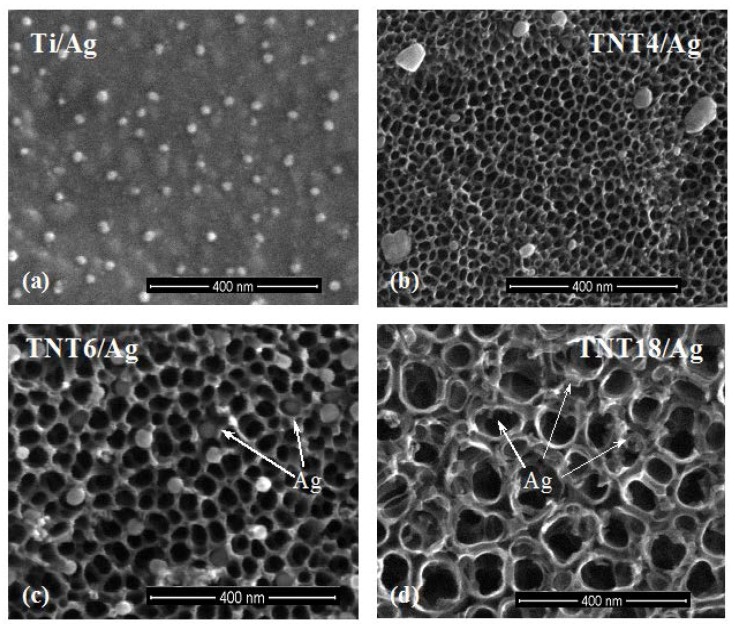
Scanning electron microscopy images of silver nanograins deposited on the surface of Ti (**a**); TNT4 (**b**); TNT6 (**c**); and TNT18 (**d**), samples, respectively (CVD, precursor: Ag(OOCC_2_F_5_), deposition temperature (*T*_D_) = 280 °C, reactor pressure (*p*) = 3 mbar, deposition time (*t*) = 30 min, mass of the precursor (*m*) = 5 mg, substrate: Titanium foil (99.6% Ti, 0.20 mm thick, STREM)).

An analysis of SEM images showed that uniform Ag films, formed on the surface of Ti substrates, are composed of dispersed grains of diameters *d*_Ag_ = 35–40 nm (Figure 1a, Table 2). The dispersed Ag grains of *d*_Ag_ = 45–65 nm were also deposited on the surface TNT4 layers (Figure 1b, Table 2). The increase in the diameter of the TiO_2_ nanotubes caused the Ag grains (*d*_Ag_ = 30–45 nm) to be mainly placed inside them (Figure 1c, Table 2). On the surface of TNT18/Ag, highly dispersed silver nanograins of diameters *d*_Ag_ ≥ 15 nm were located on the surface and inside the nanotubes (Figure 1d, Table 2), which is in agreement with reports in previously published literature [11,17,26]. The change in the precursor weight influences the density of Ag grains dispersed on the TNT substrates, but not their size and location.

**Figure 2:**
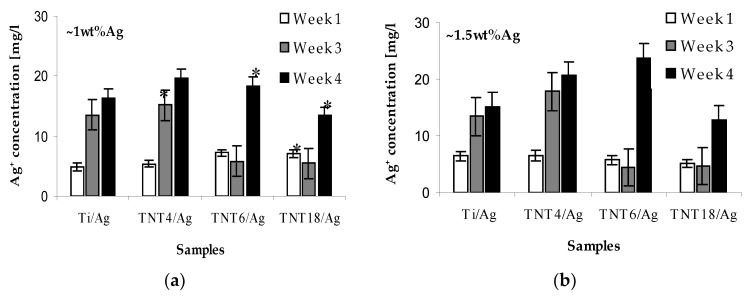
Silver ions release from TNT/Ag coatings containing 1wt% Ag (**a**) and 1.5wt% Ag; (**b**) immersed in phosphate-buffered saline (PBS) solutions. Asterisk indicates significant differences between tested and control surfaces (* *p* ≤ 0.05).

The release effects of silver ions from the TNT/Ag samples immersed in phosphate-buffered saline (PBS) solutions have been estimated using inductively coupled plasma mass spectrometry (ICP-MS). The obtained results are presented in Figure 2, as a function of immersion time.

After the first week, the concentration of the released Ag^+^ in PBS solutions was 5–8 µg/L, similar to all studied TNT/Ag nanocomposite samples. After three and four weeks, the concentration of the Ag^+^ released in PBS solutions changed, depending on the type of TNT/Ag nanocomposite coatings. For TNT4/Ag, it increased up to ca. 16–18 µg/L and 20–22 µg/L, respectively, which is higher than for the Ti/Ag reference sample (Figure 2). In the case of TNT6/Ag and TNT18/Ag coatings, for which Ag grains are mainly incorporated inside of the nanotubes, the silver ions release processes are impaired. After three weeks, a decrease in the Ag^+^ concentration in the PBS solution below 5 µg/L was noticed. A rapid increase in the release of silver ions for the above mentioned TNT/Ag layers was noticed after four weeks (Figure 2).

### 2.2. Antibacterial Activity of TNT/Ag Coatings

**Figure 3:**
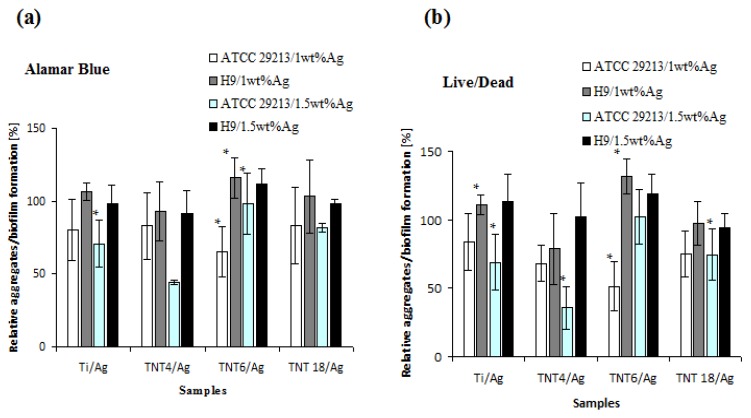
*Staphylococcus aureus* aggregates/biofilm formation on the surfaces of Ti/Ag and TNT/Ag coatings studied by (**a**) Alamar Blue staining and (**b**) Live/Dead BacLight Bacterial Viability kit. The results are presented as the mean percentage of metabolically active/live bacteria reclaimed after 24 h from Ti/Ag and TNT/Ag surfaces, compared to bacteria reclaimed from the conventional (unmodified) Ti surface (control considered as 100%). Asterisk indicates significant differences between tested and control surfaces (* *p* ≤ 0.05).

The results of antibacterial activity (considered as the inhibition of biofilm formation) studies of TNT/Ag coatings against *Staphylococcus aureus* bacteria (ATCC 29213 and H9 [22,30]), determinated using Live/Dead and Alamar Blue methods, are presented in Figure 3. An analysis of these data revealed that a clear inhibitory effect was observed for the Ti/Ag and TNT4/Ag systems against the *S. aureus* reference strain, reaching an average of 22.9–31.1% and 26.5–64.5% of the inhibition of biofilm formation, respectively. The increase in the nanotube diameter (TNT6/Ag) contributes to Ag grain localization inside of the nanotubes, influencing the reduction of the antibacterial activity of this coating (Figure 3). Moreover, TNT6/Ag nanocomposite layers loaded with 1wt% of silver grains revealed a significantly stronger inhibitory effect against the *S. aureus* ATCC 29213 biofilm (34.5% (* *p =* 0.0157) and 48.6% (* *p =* 0.0008), tested by Alamar Blue and Live/Dead, respectively) in comparison to layers containing 1.5wt% Ag (8.5% and 5.9%, tested by Alamar Blue and Live/Dead, respectively). Inhibitory effects against reference staphylococci were also observed for TNT18/Ag coatings, achieving about 25% in both methods used (the differences significant only for 1.5wt% Ag tested by Alamar Blue staining, * *p =* 0.0008); however, in this case, the anti-biofilm properties of this layer did not depend on the content of silver grains. Interestingly, neither the presence of silver grains nor nanotubes was affected biofilm formation by the *S. aureus* H9 clinical strain.

### 2.3. Fibroblasts Cells Adhesion and Proliferation

**Figure 4:**
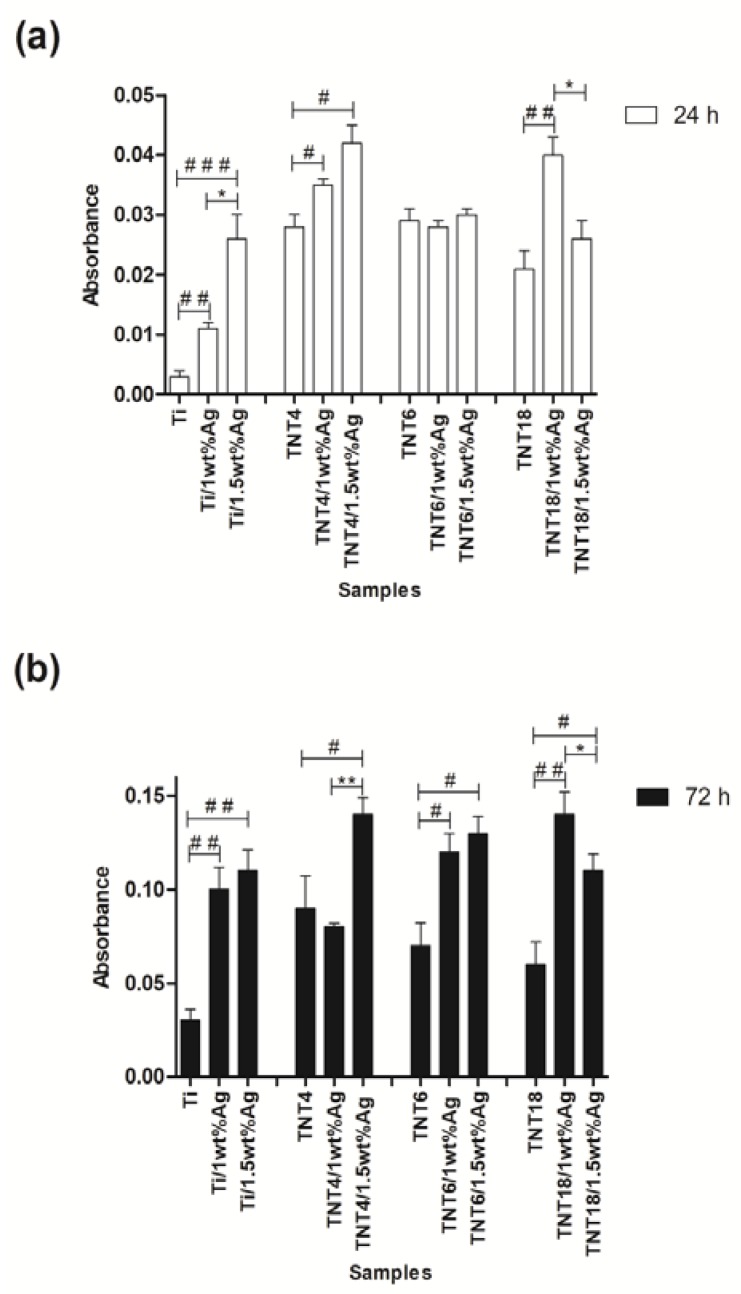
Effect of TiO_2_ nanotube coatings enriched with the different concentrations of silver nanoparticles (1wt% and 1.5wt%; TNT/Ag) on the L929 cell adhesion (after 24 h; (**a**)) and proliferation (after 72 h; (**b**)) on the nanotube surface detected by the MTT assay. The absorbance values are expressed as means ± standard error mean (S.E.M.) of three experiments. Asterisk indicates significant differences between the cells incubated with the respective nanotubes coating doped by different concentrations of Ag (1% vs. 1.5%; * *p* < 0.05, ** *p* < 0.01). Hash mark indicates significant differences between the cells incubated with TiO_2_ nanotubes or Ti plates (TNT or Ti) in comparison to the cells incubated with respective TNT or Ti coatings doped by silver nanoparticles (TNT/Ag or Ti/Ag; # *p* < 0.05, ## *p* < 0.01, ### *p* < 0.001).

The influence of the structural and morphological changes of the TNT/Ag nanocomposite coatings on the L929 murine fibroblasts adhesion (after 24 h), in comparison to proliferation (after 72 h) was estimated based on the results of the MTT assay (Figure 4a,b). Figure 4a shows that in the case of Ti/Ag composites, an increase in cell adhesion with a higher concentration of silver nanoparticles was observed (ca. 1wt% vs. 1.5wt%; * *p* < 0.05). However, for TNT coatings, consisting of TiO_2_ nanotubes with a diameter of ~100 nm (TNT18/Ag, amorphous/anatase system), the lower concentration of silver nanoparticles induced a greater cell adhesion (* *p* < 0.05 for ca. 1wt% Ag vs. 1.5wt% Ag). For TNT4/Ag nanolayers (amorphous, *d*_TNT_ = 20–30 nm), both concentrations of silver nanoparticles (ca. 1wt% Ag and 1.5wt% Ag) had a similar impact on the cell adhesion, which was higher in comparison to TNT layers without Ag (* *p* < 0.05). The layers composed of nanotubes with a diameter of 30 nm (TNT6/Ag) showed no differences in the absorbance between nanocomposites containing ~1wt% and ~1.5wt% of silver grains. As can be seen in Figure 4b, the coatings of TNT/Ag and Ti/Ag composites generally induced a greater proliferation of L929 fibroblasts in comparison to the samples not coated with silver. Furthermore, as in the case of cell adhesion, we noticed that for the nanotubes with the largest diameter (TNT 18; *d*_TNT_ = 100–150 nm), a higher concentration of silver nanoparticles (ca. 1.5wt% Ag) caused a lower cell proliferation (* *p* < 0.05). On the other hand, in the nanotubes with the smallest diameter (TNT4; *d*_TNT_ = 20–30 nm), the higher concentration of silver nanoparticles (ca. 1.5wt% Ag) resulted in a greater proliferation of fibroblasts compared to the TNT4/Ag (ca. 1wt% Ag) (* *p* < 0.01).

### 2.4. Morphology of Adherent Fibroblasts

**Figure 5:**
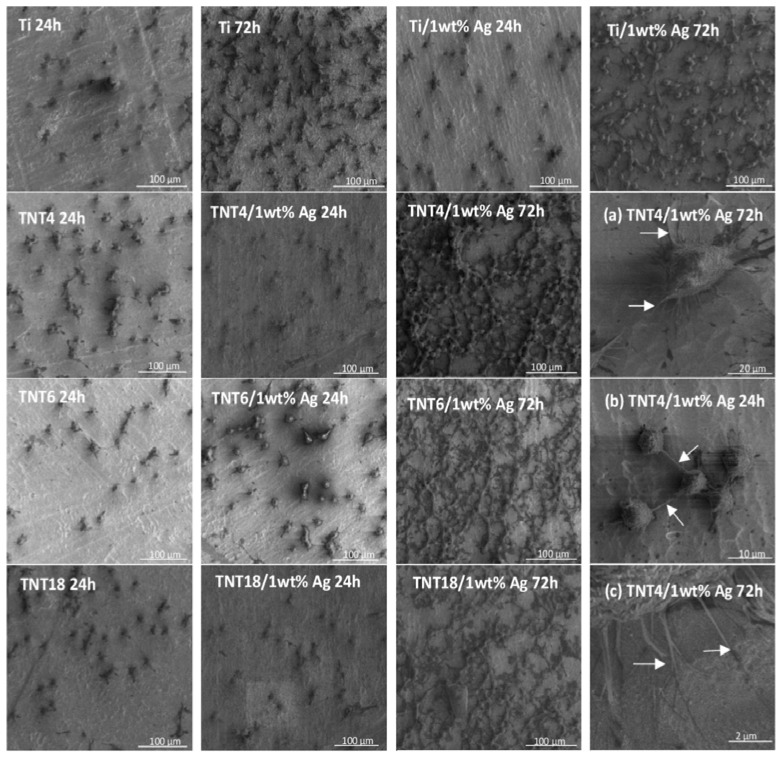
Results of SEM studies of the L929 murine fibroblasts adhesion (24 h) and proliferation (72 h) on the titanium substrate (Ti); titanium substrate deposited by silver nanoparticles (Ti/1wt% Ag); and TNT4, TNT6, and TNT18 coatings loaded or not loaded with silver nanoparticles (TNT/Ag, TNT, respectively). The arrows in Figure 5**a**–**c** indicate the filopodia spread between fibroblast (**b**) and the filopodia which attached the cells to the TNT4/1wt% Ag coating’s surface (**a**,**c**).

Analyzing the SEM images, we noticed that the fibroblasts cultivated on the pure and enriched with silver nanoparticles TNT surface coating effectively attach to the plate’s surface (Figure 5). The number of cells cultured for 72 h and attached to the surface of the plate is significantly higher in comparison to the same specimens incubated with cells for 24 h. Moreover, the fibroblasts incubated on plates for 72 h were crowded and formed networks due to the overgrowth of cells, which indicates that the tested plates could contribute to the proliferation of the cells. Our results also showed that the fibroblasts cultivated on the nanotubes started to form filopodia which attached the cells to the plate’s surface, and which spread between fibroblasts (Figure 5a–c).

### 2.5. Effect of TNT on the Synthesis of PGE_2_ and TNF-α by PBMCs

**Figure 6:**
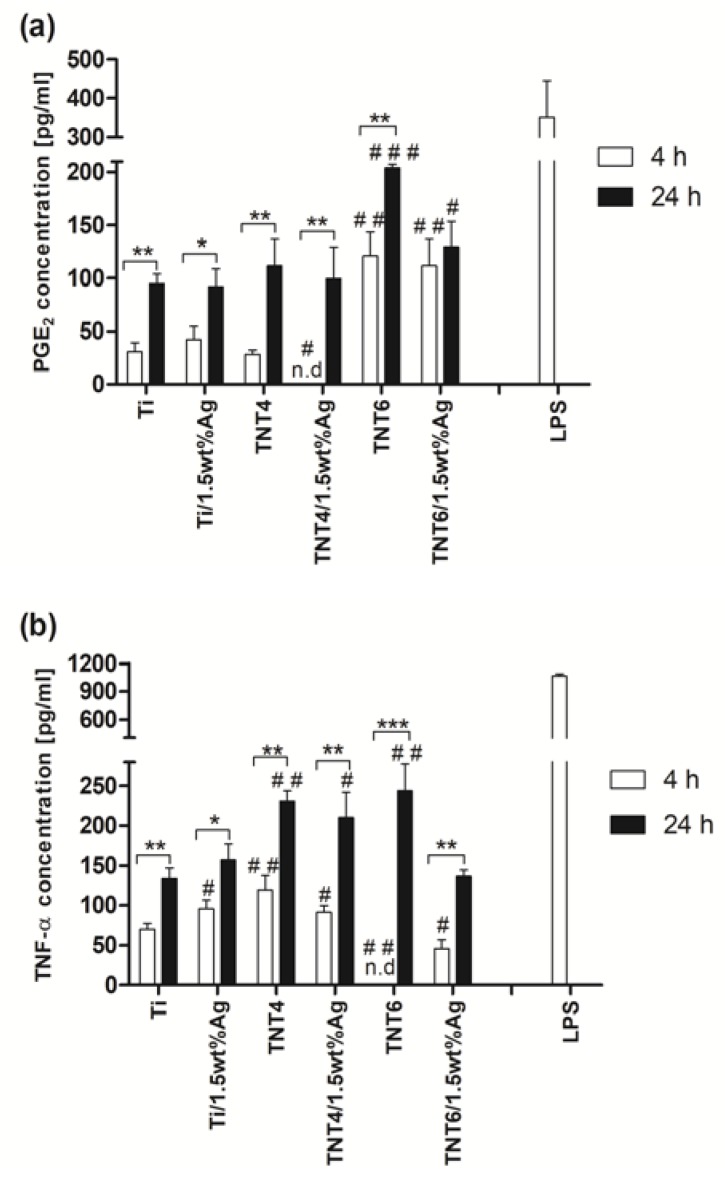
The concentration of prostaglandin E_2_ (PGE_2_; **a**) or tumor necrosis factor α (TNF-α; **b**), in the final supernatants harvested after the incubation of peripheral blood mononuclear cells (PBMCs) with the TNT/Ag coatings, detected by the ELISA assay. Levels of PGE_2_ and TNF-α were assessed for PBMCs incubated for 4 h and 24 h with titanium substrate (Ti), titanium substrate enriched with silver nanoparticles (Ti/1.5wt% Ag), TNT4, and TNT6 coatings loaded or not loaded with silver nanoparticles (TNT/1.5wt% Ag or TNT, respectively). PBMCs stimulated with LPS (1 µg/mL) for 4 h were used as a positive control for assessing the PGE_2_ and TNF-α release capacity of cells. Data are shown as means ± standard error mean (S.E.M.) of three independent experiments with two wells each. Asterisk indicates significant differences between the fibroblasts incubated with the respective biomaterial samples for 4 h (white column) in comparison to the 24-h incubation time (black column; * *p* < 0.05, ** *p* < 0.01, *** *p* < 0.001). Hash mark denotes significant differences between the cells incubated with titanium substrate (Ti) for 4 h or 24 h compared to the cells incubated with TNT, TNT/Ag, or Ti/Ag for the same period (# *p <* 0.05, ## *p <* 0.01, ### *p <* 0.001); n. d. — not detected.

The cellular activation was evaluated through the presence of tumor necrosis factor ᾳ (TNF-α) and prostaglandin E_2_ (PGE_2_), which influence the effect of specific functions within the inflammatory or wound healing processes. The immunological tests were done using PBMCs isolated from rats. Since we have observed that nanotubes with smaller diameters (TNT4, *d*_TNT_ = 20–30 nm and TNT6 *d*_TNT_ = 30–45 nm), and among them those with a higher concentration of silver nanoparticles (ca. 1.5wt% Ag), generally caused greater L929 cell proliferation, we decided to investigate their effect on the PGE_2_ and TNF-α release from PBMCs. The results indicate that both PGE_2_ and TNF-α levels in culture medium increased with time for all of the tested substrates, except for the PGE_2_ analysis for TNT6/Ag plates (ca. 1.5wt% Ag) (Figure 6a,b).

The concentration of both PGE_2_ and TNF-α in all tested supernatants was lower in comparison to the one determined in the supernatants aspirated after the stimulation of cells with lipopolisaccharide (LPS) (352 ± 94 pg/mL for PGE_2_ and 1066 ± 21 pg/mL for TNF-α; * *p <* 0.001). LPS-stimulated PBMCs (1 µg/mL for 4 h) were used as a positive control for assessing the cytokine and prostaglandin release capacity of cells. As can be seen in Figure 6a, after a 4-h incubation of cells with substrates, the greatest PGE_2_ level was noticed in the case of TNT6 (121 ± 22 pg/mL) and TNT6/1.5wt% Ag (111 ± 25 pg/mL) specimens. These values were higher compared to the one observed for the cells incubated with pure titanium plates (Ti; 30 ± 8 pg/mL; * *p <* 0.01). On the other hand, the lowest concentration was observed in the supernatants harvested after the incubation of PBMCs with TNT4/1.5wt% Ag (not detected; * *p <* 0.05). In the case of a 24-h incubation time, the PGE_2_ level was only higher when the PBMCs were incubated with TNT6 (204 ± 3 pg/mL; * *p <* 0.001) and TNT6/1.5wt% Ag (130 ± 24 pg/mL; * *p <* 0.05) in comparison to the one observed for the cells incubated with pure titanium plates (95 ± 9 pg/mL).

In contrast to the PGE_2_ results, TNF-α concentration analysis after 4-h incubation revealed the highest level of cytokine during the incubation of cells with nanotubes of a smaller diameter: TNT4 (120 ± 17 pg/mL; ** *p <* 0.01) and TNT4/1.5wt% Ag (91 ± 9 pg/mL; * *p <* 0.05) compared to Ti substrates (70 ± 6 pg/mL), whereas incubation with TNT6 (not detected; * *p <* 0.01) and TNT6/1.5wt% Ag (46 ± 11 pg/mL; * *p <* 0.05) decreased the secretion of cytokine (Figure 6b). Surprisingly, a longer incubation time resulted in higher TNF-α production, not only in the case of TNT4 (231 ± 12 pg/mL; ** *p <* 0.01) and TNT4/1.5wt% Ag (210 ± 31 pg/mL; * *p <* 0.05), but also when PBMCs were incubated with TNT6 (244 TNT4 ± 34 pg/mL; ** *p <* 0.01) when comparing these results with the cells incubated with pure titanium specimens (134 ± 13 pg/mL).

## 3. Discussion

**Figure 7:**
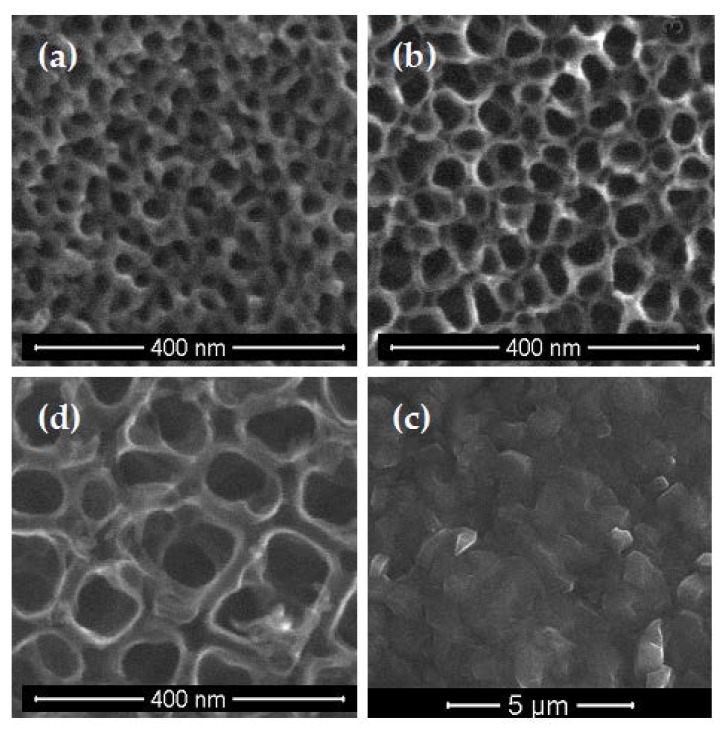
SEM images of TNT4 (**a**); TNT6 (**b**); TNT18 (**d**) heated at 573 K; and TNT6 (**c**) heated at 623 K.

According to our earlier works, the anodization process of the titanium substrate surface at different voltages leads to the formation of TNT layers with different nanotube diameters, structures, and packing densities [10,31]. The formation of the nanotubular architecture was observed both on the flat substrates, as well as on the surface of 3D implants. The layers produced at potentials 4 V (TNT4), 6 V (TNT6), and 18 V (TNT18) have been selected for our works on the formation of TNT/Ag nanocomposites. With this choice, the possible correlations between the TNT coatings structure and their bioactivity have been considered [10]. The use of the CVD method, in order to enrich TNT coatings with the dispersed silver grains, required solving the following problems: (a) the assessment of structural and morphological stability of TNT coatings during their heating up to 573 K; and (b) the choice of CVD precursor for the deposition of dispersed Ag grains below 573 K. Earlier works revealed that the amorphous TNT layers heated up to 1073 K were still amorphous below 553 K [22,30]. The results of our experiments proved that the morphology and amorphousness of TNT4 and TNT6 samples did not change during their heating up to 613 K, while beyond this temperature, the tubular architecture of TNT layers was destroyed (Figure 7). An analysis of the Raman spectra of the TNT18 sample, heated up to 573 K, revealed the formation of the layer, composed of TiO_2_ anatase nanotubes (Figure S3).

Among the CVD silver precursors that we studied, the properties of silver(I) pentafluoropropionate (Ag(OOCC_2_F_5_)) appear to be suitable for use in the preparation of TNT/Ag nanocomposites [32,33,34,35]. A simple and inexpensive synthesis, and good volatility, thermal stability of vapors, and deposition of pure and dispersed silver grains below 573 K were the main factors leading to the choice of this compound. The results of XRD and XPS studies confirm the presence of metallic Ag grains on the surface of TNT films. An analysis of SEM images showed that the size and the position of Ag grains changed with the increase in the titania nanotube diameter (Figure 1). On the surface of TNT4 layers (*d*_TNT_ = 20–30 nm), silver grains of *d*_Ag_ = 45–65 nm have been primarily deposited on the TNT matrix surface. The increase in the TiO_2_ nanotube diameter up to *d*_TNT_ = 30–45 nm (TNT6/Ag) caused Ag grains of *d*_Ag_ = 35–45 nm to be mainly placed inside the nanotubes. On the surface of large TiO_2_ nanotubes (*d*_TNT_ = 100–150 nm, TNT18/Ag), silver nanograins of *d*_Ag_ > 15 nm were located on the top edges and walls of tubes.

According to the data presented in Figure 2, the amount of released silver ions after the first week (5–10 µg/L) was lower than for other reported nanocomposite materials [22,27,28], and was independent of the silver concentration on the TNT coating surface (1–1.5wt%). After the following three to four weeks, the concentration of the released Ag^+^ in PBS solutions changes, and depends on the type of TNT/Ag nancomposite coating. For TNT4/Ag systems, it increases up to ca. 16 µg/L and ca. 20 µg/L, respectively, and was higher in comparison to the reference sample (15–18.5 µg/L) (Figure 2). The slight differences, which were observed between the TNT4/Ag and Ti/Ag samples, may be due to the presence of silver grains only on the substrate surface. Ag ion release processes are similar, but the presence of adsorbed H_2_O molecules on the TNT4/Ag surface (Figure 2) promotes oxidation processes resulting in a higher concentration of Ag^+^ in comparison to Ti/Ag. In the case of TNT6/Ag and TNT18/Ag coatings, the growth of silver grains proceeds mainly inside the nanotubes. Silver ion release processes are impaired (4–6 µg/L), resulting in the inhibition of their concentration in PBS solution after three weeks. The rapid increase in the silver ion release was observed for TNT/Ag nanocomposite layers after four weeks, reaching levels of almost 24 µg/L, but were still lower in comparison to previously reported ones [22,27,28]. The effect of the initial decline and subsequent rapid growth of the silver ions release rate from the polyamide/silver (PA/Ag) composites was also noticed by Kumar et al. [36]. According to their explanation, the initial high release rate of Ag ions is related to the oxidation processes of the particles deposited on the coating surface. To release Ag^+^ ions from the interior part of the layer, water has to cross the diffusion barrier, which may be associated with changes in the structural state of the material, and in the consequence with the oxidation and migration of Ag ions from the interior part of the layer. This effect influences both the long term antibacterial activity of these materials and the safe dose of silver for humans [37].

*Staphylococcus aureus*, as one of the clinically relevant pathogens in implant-related infections, was chosen as a model to test the anti-biofilm activity of Ag-modified titanium surfaces. Because of the fact that microbial cells in the biofilm exhibit different levels of metabolic activity: Metabolically active cells, starving cells, dormant cells, viable-but-nonculturable cells, persisters, and finally dead cells [38], at least two different methods should be used to assess a microbial biofilm. Therefore, a Live/Dead BacLight Bacterial Viability kit was used to distinguish live and dead bacteria based on cell membrane integrity and Alamar Blue staining was employed to check microbial cell metabolic activity. The obtained results showed the inhibitory effect of the TNT4/Ag system, formed by the deposition of the large and dispersed silver grains on the surface of TNT coatings, consisting of small diameter nanotubes. This inhibitory effect was also observed in our earlier works concerning TNT layers made of densely packed nanotubes of *d*_TNT_ = 20–30 nm [10,39]. A weak inhibitory effect was also observed for TNT18/Ag coatings enriched by Ag grains of a diameter lower than 15 nm. Such an effect can be seen as the result of two combining factors, i.e., (a) the anatase form of TiO_2_ nanotubes; and (b) the presence of small silver grains, located on the top edge of each nanotube and also on their walls. This effect was also strain-dependent, indicating that the *S. aureus* ATCC 29213 reference strain was susceptible to the biostatic/biocidal activity of the Ag presence as the clinical *S. aureus* H9 strain. Because of the multidirectional activity of Ag nanoparticles, including the increase in the cell wall permeability, the damage of membrane transport, the nucleic acid synthesis, the protein production, and their enzymatic activity, the development of a total resistance to silver is rather unlikely [40]. However, clinical strains could be less sensitive, due to possible long-term contact with biomaterials containing Ag nanoparticles (e.g., dressings).

Due to the fact that silver exhibits cytotoxicity to some cells at specified concentrations [41], in our experiments, the TNT/Ag or/and Ti/Ag nanocomposites did not inhibit the growth of L929 fibroblasts (Figure 4). The larger size of Eukaryotic cells in comparison to prokaryotic cells means that Eukaryotic cells are more susceptible to a silver ion attack. Moreover, these cells also exhibit more structural and functional redundancy than prokaryotic cells. Therefore, higher silver ion concentrations are required to achieve comparable toxic effects, relative to bacterial cells [42]. Similarly, Liao et al. [43] and Chang et al. [44] did not observe the inhibition of growth of human gingival fibroblasts (HGF) on the Ti/Ag and TiO_2_/Ag coatings, compared to control specimens. Moreover, in our studies, the presence of silver nanoparticles led to a greater proliferation of cells on their surface. Huang et al. [45] also demonstrated that Ti plates coated with Ag showed better HGF cell viability and proliferation than uncoated samples. The data presented in Figure 5 suggests that the coating morphology, its structure, and concentration of silver nanoparticles dispersed on the TNT layer surface (ca. 1wt% Ag vs. 1.5wt% Ag) are the main factors affecting the adhesion (Figure 5a) and proliferation (Figure 5b) of L929 fibroblasts.

Lu et al. [46] have tested the biocompatibility of Ti implants incorporated with different concentrations of AgNPs (0.5, 1, 1.5, 2 M). For all tested concentrations, the beginning of the osteoblast adhesion on the coatings was observed after one day of culture and spread well until seven days of culture. However, after this, the inhibitory effect of 1 M Ag on cell proliferation was observed, what may suggest that coatings with low concentrations of silver were more favorable for osteoblast growth. Lu et al. [45] investigated Ti implants, whereas in our study, we tested TNT coatings with different diameters of TiO_2_ nanotubes. Lan et al. [40] proved that TNT/Ag coatings exhibited a monotonically increasing trend in MRC-5 human fibroblast cell line proliferation with a decreasing nanotube diameter. According to these data, fibroblast adhesion and proliferation showed an obvious diameter-dependence behavior of titania nanotubes enriched with silver. However, the concentration of silver nanoparticles used by these authors was different than in our case.

An analysis of scanning electron microscopy images confirmed the results of the MTT assay (Figure 5). The number of fibroblasts cultured for 72 h is significantly higher compared to the same specimens incubated with cells for 24 h. Moreover, the cells cultivated on the nanotubes started to form a filopodia spread between fibroblasts, which also attached the cells to the plate (Figure 5a–c). Similarly, Swan et al. [47] noticed the filopodia extending processes in the nanopores, which can increase the osteoblast adhesion. These thin membrane protrusions have a sensory activity in cells and they are sensitive to nanotopography, causing changes in cell morphology by filopodia guidance [48,49]. The tested TNT coatings enriched with silver nanoparticles demonstrate biocompatible properties, associated with a favorable cellular interaction with their surface. This phenomenon is important for the long-term success of the implant incorporation [50]. Fibroblasts are the most important cells in connective tissue, as one of the main components of peri-implant soft tissue, which is crucial to the formation of the peri-implant mucosal seal and helps to prevent epithelial ingrowth [51]. In future experiments, we are going to investigate the impact of TNT/Ag on the viability and proliferation of other cells, such as osteoblasts, to verify the biocompatible properties of the tested nanotubes. An explanation of how osteogenic cells interact with TNT/Ag in vitro would be helpful to design better implants for bone regeneration and implant integration in vivo.

According to earlier reports, the TNT layer formation improved the cellular behaviors including proliferation, adhesion, and spreading [52]. Therefore, the combination of antibacterial activity (from Ag) and biointegration properties (from TiO_2_) of the TNT/Ag coating may be advantageous for medical use.

Inflammatory and wound healing cells, such as peripheral blood mononuclear cells, including lymphocytes and monocytes, release inflammatory cytokines and prostaglandins following cellular adhesion and activation [53]. Cytokines and prostaglandins create a complex network, which modulate cellular interaction, infiltration, communication, and behavior [54]. Moreover, additional activation and matrix formation perpetuates inflammatory or wound healing responses. Cytokines and prostaglandins bind to surface cellular receptors, either inducing (pro-inflammatory) or inhibiting further intracellular functions, intercellular communication, and extracellular matrix development [55,56]. For this reason, cytokines and prostaglandins are key factors in evaluating cellular activation and communication. In our study, the immunological tests were done using PBMCs isolated from rats.

The results from PGE_2_ analysis have revealed that after 4-h incubation of PBMCs with substrates, the greatest PGE_2_ level occurs in the case of TNT6 and TNT6/1.5wt% Ag specimens. These values were higher compared to the ones observed for the cells incubated with pure titanium plates. In the case of a 24-h incubation time, the PGE_2_ level was only higher when PBMCs were incubated with TNT6 and TNT6/1.5wt% Ag, in comparison to what was observed for the cells incubated with pure titanium plates (Figure 6a). The earlier works proved that, in the case of osteoblasts, surface roughness affects the cell response associated with the production of local factors such as PGE_2_ [57], stimulating osteoclast-like cell formation and bone-resorbing activity [58]. The stimulatory effect of the rougher surfaces on PGE_2_ production was greater on titanium plates than Ti6A14V alloys [59]. Moreover, Yang et al. [60] showed that TiO_2_ nanotubes stimulated fetal rat calvarial cells to release a greater amount of PGE_2_ than a mechanically treated titanium surface. In our study, we demonstrated that all tested substrate activated PBMCs release greater amounts of PGE_2_ over time. This is a desirable phenomenon since prostaglandin is believed to regulate growth factor synthesis such as transforming growth factor β1 (TGF-β1) in osteoblast differentiation [61]. Furthermore, prostaglandins have been demonstrated to stimulate both bone resorption and bone formation, but in favor of bone formation, thus increasing bone mass and bone strength [62]. In contrast to the PGE_2_ results, TNF-α concentration analysis after 4-h incubation revealed the highest level of cytokine during the incubation of cells with nanotubes of a smaller diameter (TNT4 and TNT4/1.5wt% Ag), compared to Ti substrates, whereas incubation with TNT6 and TNT6/1.5wt% Ag decreased cytokine secretion. Surprisingly, however, a longer incubation time resulted in higher TNF-α production, not only in the case of TNT4 and TNT4/1.5wt% Ag, but also when PBMCs were incubated with TNT6 (Figure 6b). Tumor necrosis factor α is a pro-inflammatory mediator, regulating a wide range of biological processes, including cell proliferation, differentiation, and apoptosis. This cytokine stimulates the release of matrix metalloproteinases (MMPs) and is secreted by the resident cells such as osteoblasts, gingival fibroblasts, and immune cells. It is also responsible for an early reaction to microbial plaque accumulation, attracting other inflammatory cells, and is a key regulator for the formation of osteoclasts and the local resorption of bone tissue, effectively forming and enlarging gaps at the prosthesis-tissue interface [63]. On the other hand, TNF-α, at low levels, might promote wound healing by indirectly stimulating inflammation and increasing macrophage growth factors. However, at higher levels, especially over prolonged periods of time, it has a detrimental effect on healing [64]. Nevertheless, in our study, we showed that, in tested substrates activating PBMCs to release greater amounts of TNF-α over time, these values were much lower compared to the LPS-stimulated cells. In order to confirm or exclude pro-inflammatory properties of the tested nanotubes in future experiments, we are going to investigate the level of other pro-inflammatory cytokines (i.e., IL-6, IL-1β) and chemokines (i.e., monocyte chemoattractant protein-1, macrophage inflammatory protein-1α) in the final supernatants harvested after the incubation of PBMCs with the specimens in the presence or absence of LPS.

## 4. Materials and Methods

### 4.1. The Fabrication of Ti/TNT Coatings and Their Characterization

Titania nanotube coatings (TNT) on the surface of the titanium foil (5 mm × 70 mm, 99.6% Ti, 0.20 mm thick, STREM) were produced by electrochemical anodic oxidation, according to the previously described method [4]. The anodization processes were carried out at potentials: 4 V (TNT4), 6 V (TNT6), and 18 V (TNT18), and at an anodization time *t* = 20 min. The morphology of the produced coatings was studied using a scanning electron microscope (SEM; Quanta 3D FEG; Carl Zeiss, Göttingen, Germany; 30 kV accelerating voltage; micrographs were recorded under high vacuum using a secondary electron detector (SE)). The structure of the produced TNT layers was analyzed using Raman spectroscopy (RamanMicro 200 PerkinElmer, Waltham, MA, USA; 200 (λ = 785 nm)) and X-ray photoelectron spectroscopy (XPS). XPS spectra were registered with monochromatized Al Kα radiation (1486.6 eV) at room temperature, using a PHI 5700/660 ESCA spectrometer (Physical Electronics, Lake Drive East, Chanhassen, MN, USA).

### 4.2. Synthesis and Characterization of TNT/Ag Nanocomposite Coatings

The fabricated TNT coatings were enriched with silver grains using the CVD method (horizontal *hot-wall* reactor) under the conditions presented in Table 3. The precursor Ag(OOCC_2_F_5_) was synthesized as reported [65,66]. The morphology of produced coatings was studied using a scanning electron microscope (Quanta 3D FEG). The structure of the produced TNT layers was analyzed using Raman spectroscopy and X-ray photoelectron spectroscopy (XPS).

The release studies of silver ions: Ti/Ag (reference sample) and TNT/Ag coatings, were immersed in phosphate buffered saline (PBS) solution at 310 K (human body temperature) for one, three, and four weeks. The silver concentration in elutes was measured by means of an inductively coupled plasma mass spectrometry device (ICP MS Agilent Technologies 7700x, Yokogawa Analytical Systems Inc., Tokyo, Japan), calibrated with five dilutions (1.00, 2.50, 5.00, 7.50, 10.00 µg/dm^3^) of the Multi-Element Aqueous CRM Environmental Calibration Standard A (VHG Labs., Manchester, NH, USA) with an original silver concentration of 10.00 ± 0.05 µg/dm^3^. Qualitative and quantitative analysis of the concentration of Ag was performed using the software MassHunter Workstation Software (Agilent Technologies, Yokogawa Analytical Systems Inc., Tokyo, Japan) for ICP-MS Version A.01.02 G7201A Build 291.22 Patch 5.

### 4.3. Antibacterial Properties of TNT/Ag Coatings

The produced TNT/Ag nanocomposite coatings on the surface of titanium foil were exposed to the *Staphylococcus aureus* ATCC 29213 reference strain and *S. aureus* H9 clinical strain, which were prepared in accordance with the previously used procedure [10]. The samples (size ca. 5 mm × 5 mm) of studied TNT/Ag layers, Ti/Ag films, and titanium (reference sample—control K1) were placed into *S. aureus* suspensions for 24 h and incubated in stable conditions at 310 K to form microbial aggregates/biofilm [10]. To evaluate aggregate/biofilm formation, a Live/Dead BacLight Bacterial Viability kit (L/D; Molecular Probes, Waltham, MA, USA), Alamar Blue (AB; BioSource, Dacula, GA, USA) staining, and the CFU method were used. For all types of titanium samples, two independent sets of experiments were prepared, each in quadrupluicate. The obtained results were presented as a percentage of metabolically active bacteria (Alamar Blue staining) or alive bacteria (Live/Dead method) reclaimed from the aggregates/biofilm formed on the titanium samples tested (Ti/Ag and TNT/Ag), calculated from the mean percentage of AB reduction ± S.D. or the mean relative fluorescence units RFU ± S.D., respectively, of the control K1 (considered as 100%) and test wells. The nonparametric Kruskal-Wallis one-way ANOVA was used to compare the differences among biomaterial samples. * *p* ≤ 0.05 was considered significant.

### 4.4. L929 Cells Culture

L929 murine fibroblast cells (American Type Culture Collection) were cultured in 25 cm^2^ cell culture flasks (Corning, NY, USA), at 310 K in a humidified atmosphere with 5% CO_2_. Culture medium consisted of complete RPMI 1640 medium, containing 2 mM l-glutamine (Sigma-Aldrich, Darmstadt, Germany), 10% heat-inactivated fetal bovine serum (FBS), 100 IU/mL penicillin, and 100 µg/mL streptomycin (PAA Laboratories GmbH, Cölbe, Germany). The culture medium was changed every three days. L929 cells were passaged when reaching 70–80% confluency.

### 4.5. L929 Cells Adhesion and Proliferation Assay on TNT

The effects of TiO_2_ nanotube coatings loaded with silver nanoparticles (TNT/Ag) on the L929 cells adhesion (after 24 h) and proliferation (after 72 h) were studied using the MTT (3-(4,5-dimethylthiazole-2-yl)-2,5-diphenyl tetrazolium bromide; Sigma Aldrich; Darmstadt, Germany) assay, as described previously [10]. Briefly, the cells at a density of 1 × 10^4^ cells/well were seeded in 24-well culture plates on the samples sterilized by autoclaving, and incubated in 1 mL of complete RPMI 1640 medium for 24 h and 72 h. After the respective incubation time, the specimens were accurately washed three times with phosphate buffered saline (PBS) and transferred to a new 24-well plate. The MTT (5 mg/mL; Sigma-Aldrich, Darmstadt, Germany) solution in RPMI 1640 medium without phenol red (Sigma-Aldrich, Darmstadt, Germany) was added to each well and the plates were incubated at 310 K for 3 h to form formazan. After aspirating the solution from each well, 500 µL of dimethyl sulfoxide (DMSO; 100% *v*/*v*; Sigma Aldrich, Darmstadt, Germany) was added into each well to dissolve the formazan crystals formed on the plate surface. After shaking for 10 min, the absorbance was measured at 570 nm with the subtraction of the 630 nm background using a Synergy HT Multi-Mode Microplate Reader (BioTek Instruments, Winooski, VT, USA). The blank groups (TNT/Ag incubated without cells) were treated with the same procedures as experimental groups. Culture medium without the TNT/Ag was used as a negative control in each experiment. All measurements were done in duplicate, in three independent experiments. The results were reported as means ± standard error mean and were analyzed by one-factor analysis of variance (ANOVA). As a post hoc test, the Tukey test was used. The significance level was set at * *p <* 0.05. The morphology changes of L929 cells grown on the surface of TiO_2_ nanotube coatings loaded with silver nanoparticles were studied using Scanning electron microscopy (SEM; Quanta 3D FEG; Carl Zeiss, Göttingen, Germany). For the SEM analysis, the samples were prepared in accordance with the previously described procedure [10]. Briefly, after the selected incubation period, the samples were washed three times with PBS to remove the non-adherent cells and fixed in 2.5% glutaraldehyde (Sigma Aldrich, Darmstadt, Germany) for a minimum of 4 h. After that, the specimens were rinsed three times with PBS and then dehydrated in a graded series of alcohol (50, 75, 90, and 100%), before being dried in vacuum-assisted desiccators overnight and stored at room temperature till SEM analysis was carried out.

### 4.6. Peripheral Blood Mononuclear Cells Isolation and Culture

Blood was sterile collected from anesthetized 10-week old male Wistar rats by cardiac puncture into a solution of K_3_EDTA (Sigma Aldrich, Darmstadt, Germany). Peripheral blood mononuclear cells (PBMCs) isolation was performed according to the density gradient centrifugation method, as we described previously [67,68]. Briefly, the whole collected blood was diluted 1:1 (*vol*/*vol*) with PBS. The diluted cell suspension was carefully layered onto the separation medium (Ficoll-Paque Plus, Amersham Biosciences, Piscataway, NJ, USA) and centrifuged (35 min, 400× *g*). After centrifugation, the PBMCs fraction was collected and washed twice with RPMI 1640 medium (RPMI-1640 with l-glutamine; Sigma Aldrich, Darmstadt, Germany), before being suspended in the complete RPMI medium, supplemented with 10% heat inactivated fetal bovine serum (FBS; PAA Laboratories GmbH, Cölbe, Germany), 100 IU/mL penicillin, and 100 µg/mL streptomycin (PAA Laboratories GmbH, Cölbe, Germany). PBMCs were cultured at 310 K in a humidified atmosphere with 5% CO_2_.

### 4.7. PBMCs Culture Condition for Enzyme-Linked Immunosorbent Assay (ELISA)

Before culturing, the count and viability of freshly isolated PBMCs were determined by trypan blue exclusion test, using the LUNA^TM^ automated cell counter (Logos Biosystems, Annandale, VA, USA). In order to evaluate the immune response, the cells were seeded on titania nanotube arrays in a 24-well plate (Corning, NY, USA). Prior to seeding, all substrates (sterilized by autoclaving) were placed in the wells. For tumor necrosis factor α (TNF-α) and prostaglandin E_2_ (PGE_2_) assessments, the cells were seeded at a density of 1 × 10^6^ cells/well in a total volume of 1 mL RPMI 1640 medium, supplemented with 10% FBS and antibiotics. The substrates were incubated with cells at 310 K for 4 h or 24 h in an incubator providing a humidified atmosphere containing 5% CO_2_. Additional, PBMCs without the TNT were stimulated with 1 μg/mL lipopolysaccharide (LPS extracted from *Escherichia coli* 0111:B4, Sigma Aldrich, Darmstadt, Germany) for 4 h as a positive cellular control, in order to evaluate the levels of TNF-α and PGE_2_ in the final supernatants harvested after the incubation of cells with TNT. LPS was diluted in a pyrogen-free saline and added to the culture in a volume of 100 µL. The final supernatants from each experiment were aspirated, centrifuged for 5 min at 1000× *g*, and stored at 223 K. The final analysis for the release of TNF-α and PGE_2_ from PBMCs (mostly monocytes) attached to the surface of the plates was made in duplicate. The concentration of TNF-α and PGE_2_ was determined by standard sandwich ELISA kits from R&D Systems (Minneapolis, MN, USA, cat. no. RTA00 and KGE004B, with a detection limit of less than 5 pg/mL and 31 pg/mL, respectively), according to the manufacturer’s instructions. Colorimetric changes in the assays were detected using a Synergy HT Multi-Mode Microplate Reader (BioTek Instruments, Winooski, VT, USA).

## 5. Conclusions

The advantage of the CVD method, which was applied to enrich the titania nanotube coatings (TNT) with silver nanograins, is the possibility to control the size of Ag grains and their location on the surface of the TNT matrix. The results of our works revealed that TiO_2_ nanotube diameters also seem to be significant for the above mentioned control possibility.

The results of silver ion release from the surface of TNT/Ag coatings indicate the promising properties of TNT4/1.5wt% Ag layers, in which dispersed Ag grains are only located on the surface. This type of coating revealed the best antibacterial responses and also characterizes the appropriate biocompatibility, promoting fibroblast adhesion and proliferation. Moreover, the lower PGE_2_ secretion of this sample was observed. Good antibacterial activity was also noted for the TNT6/1 wt% Ag sample. However, in this case, it depends on the Ag particle location, i.e., on tube top edges (the increase of silver ions concentration in PBS solution released in the first week) or inside of them (the rapid increase of Ag^+^ concentration after four weeks). In general, low levels of TNF-α secretion and simultaneous PGE_2_ secretion suggest that TNT6/1wt% Ag samples may perform better for biointegration.

In summary, the produced TNT/Ag nanocomposite coatings revealed better properties for medical applications, in comparison to titania nanotube layers. The combination of antibacterial properties of the TNT/Ag nanocomposite, coming from the presence of silver nanograins, and biocompatibility given by TiO_2_, turns out to be promising for the medical applications of these materials.

## Figures and Tables

**Table 1:** X-ray photoelectron spectroscopy peak positions for TNT/Ag coatings (TiO_2_ nanotubes were produced at 4 V (TNT4), 6 V (TNT6), and 18 V (TNT18)) on the surface of titanium foil (99.6% Ti, 0.20 mm thick, STREM).

Sample	Ti^4+^		O^2−^	OH^−^	H_2_O	Ag	
	Ti (2p_3/2_) (eV)	Ti (2p_1/2_) (eV)	O (1s) (eV)	O (1s) (eV)	O (1s) (eV)	Ag (3d_5/2_) (eV)	Ag (3d_5/2_) (eV)
TNT4/Ag	458.9	464.7	530.5 (58%)	532.0 (29%)	533.2 (13%)	368.5	374.5
TNT6/Ag	459.3	465.1	530.1 (71%)	531.8 (22%)	533.0 (7%)	368.8	374.9
TNT18/Ag	459.5	465.3	530.3 (54%)	531.7 (13%)	532.7 (33%)	368.0	373.9

**Table 2:** Diameters of titania nanotubes and average diameters of silver nanoparticles, which were found on the surfaces of TNT/1wt% Ag and TNT/1.5wt% Ag nanocomposite coatings.

Sample	*d*_TNT_ (nm)	*d*_Ag_ (nm)
Ti/Ag	-	35–40
TNT4/Ag	20–30	45–65
TNT6/Ag	35–45	30–45
TNT18/Ag	100–150	≥15

**Table 3:** Summary of CVD conditions for the deposition of silver nanograins.

Precursor	Ag(OOCC_2_F_5_)
Precursor weight (mg)	5 and 10
Vaporization temperature (*T*_V_) (K)	513
Carrier gas	Ar
Total reactor pressure (*p*) (mbar)	3.0
Substrate temperature (*T*_D_) (K)	553
Substrates	Ti/TNT
Deposition time (min)	30

## Supplementary Materials

The following are available online at http://www.mdpi.com/2079-4991/7/9/274/s1, Figure S1: X-ray diffraction pattern of silver grains deposited on the surface of TNT6; Ag: 38.1 (111), 44.6 (200), 64.7 (220), 77.5 (311) and 81.5 (222), Ti: 35.1 (100), 38.4 (002), 40.2 (101), 53.0 (102), 70.7 (103), 76.3 (112) and 82.3 (004) (results of these investigations indicate on the amorphousness of the TNT6 coating, CVD, *T*_D_ = 553 K, *p* = 3 mbar, *t* = 30 min., *m* = 10 mg), Figure S2: Deconvolution of O(1s) peak in the XPS spectrum of TNT/Ag coatings. Additionally, Ti(2p) and Ag(3d) XPS peaks are presented, Figure S3: Raman specta of TNT samples heated up to 573 K.
