# Supplementary material for: Biocompatibility of Titania Nanotube Coatings Enriched with Silver Nanograins by Chemical Vapor Deposition
Source: Nanomaterials (Basel). 2017 Sep 15;7(9):274. doi: 10.3390/nano7090274 (PMC5618385; doi:10.3390/nano7090274)
Supplement: Supplementary file 1 [file nanomaterials-07-00274-s001.pdf]

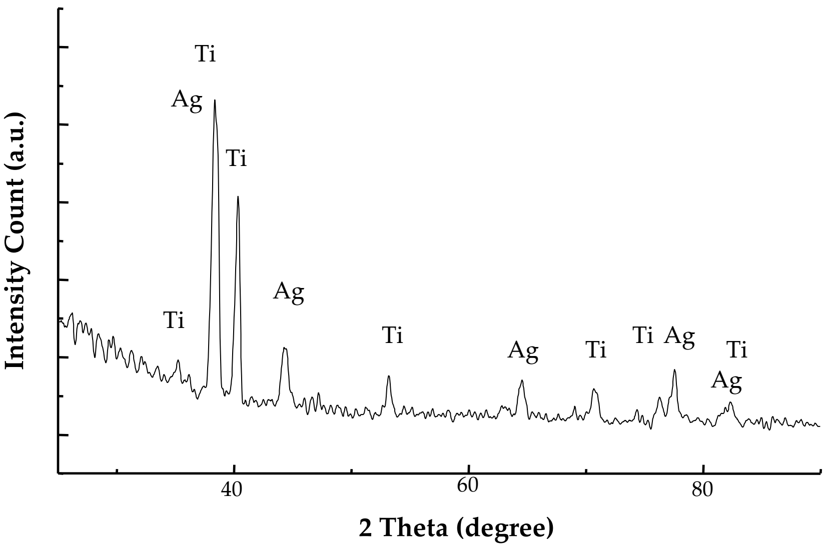

Figure S1. X-ray diffraction pattern of silver grains deposited on the surface of TNT6; Ag: 38.1 (111), 44.6 (200), 64.7 (220), 77.5 (311) and 81.5 (222), Ti: 35.1 (100), 38.4 (002), 40.2 (101), 53.0 (102), 70.7 (103), 76.3 (112) and 82.3 (004) (results of these investigations indicate on the amorphousness of the TNT6 coating, CVD, T_D = 553 K, p = 3 mbar, t = 30 min., m = 10 mg).

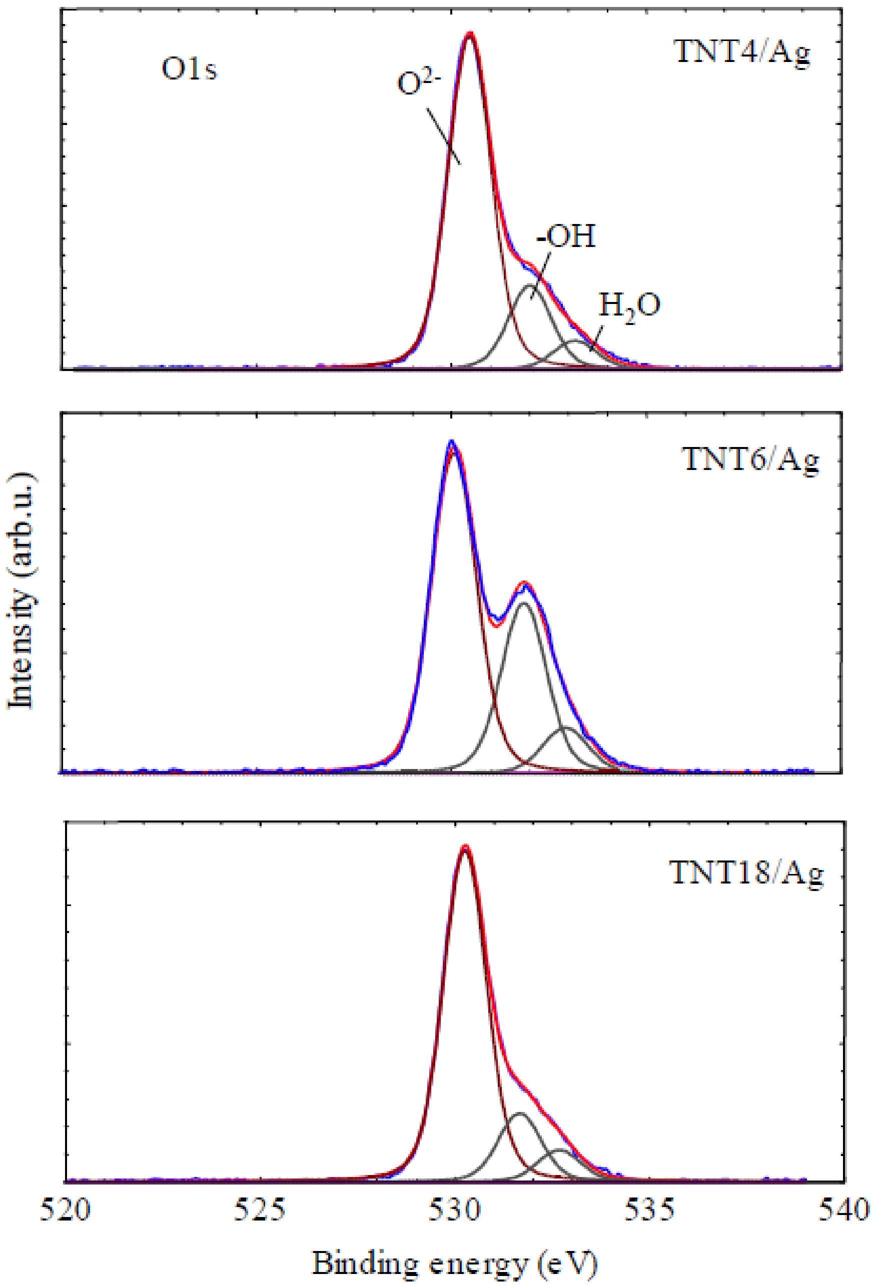

Figure S2. The deconvolution of O(1s) peak in the XPS spectrum of TNT/Ag coatings.

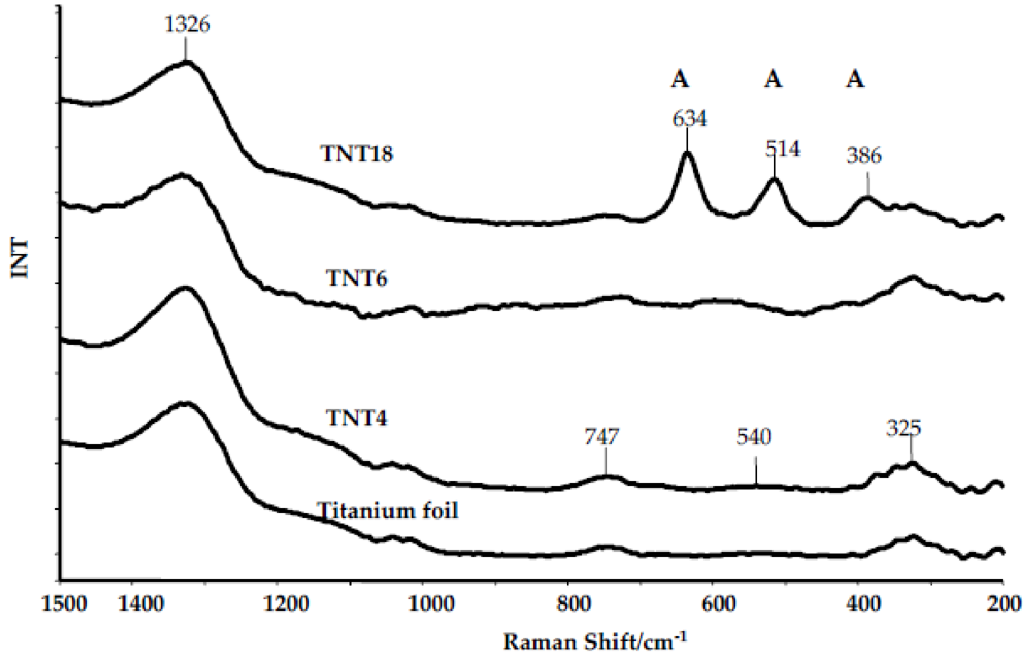

Figure S3. Raman specta of TNT samples heated up to 573 K.
